# Bovine mastitis: prevalence, risk factors and isolation of *Staphylococcus aureus* in dairy herds at Hawassa milk shed, South Ethiopia

**DOI:** 10.1186/s12917-016-0905-3

**Published:** 2016-12-03

**Authors:** Rahmeto Abebe, Hagere Hatiya, Mesele Abera, Bekele Megersa, Kassahun Asmare

**Affiliations:** 1 School of Veterinary Medicine, Hawassa University, P.O.Box 05, Hawassa, Ethiopia; 2 Animal and Fisheries Resources Development Bureau of Southern Nations, Nationalities and Peoples’ Regional State, P.O.Box 309, Hawassa, Ethiopia

**Keywords:** CMT, Dairy herds, Mastitis, Risk factors, *S. aureus*

## Abstract

**Background:**

Mastitis is a disease of major economic importance in dairy industry worldwide. It is of particular concern in developing countries like Ethiopia, where milk and milk products are scarce. The objectives of the study were to estimate the prevalence of mastitis, identify the cow-and herd-level potential risk factors and isolate *Staphylococcus aureus*, one of etiological agents for contagious mastitis, from cows positive for mastitis. A total of 529 lactating cows selected randomly from 95 herds were screened by California mastitis test (CMT) for sub-clinical mastitis. Also 172 milk samples collected from CMT positive cows were cultured for isolation of *S. aureus*.

**Results:**

Based on CMT result and clinical examination, the prevalence of mastitis at herd-level was 74.7% (95% CI: 64.5, 82.8). The corresponding cow-level prevalence was 62.6% (95% CI: 58.3, 66.7), of which 59.2 and 3.4% were sub-clinical and clinical mastitis cases, respectively. *S. aureus* was isolated from 51.2% of the milk samples cultured and 73.2% of the herds affected with mastitis. In the multivariable logistic regression model, the herd-level factors significantly associated (*p* < 0.05) with the presence of mastitis were herd size, bedding material, and milking mastitic cows last, while at cow-level, breed, parity, stage of lactation, udder and leg hygiene, and teat end shape were noted to have a significant effect on mastitis occurrence.

**Conclusion:**

The very high prevalence of mastitis, more importantly the sub-clinical one, in the herds examined revealed the huge potential economic loss the sector suffers. Perhaps this was attributed to lack of implementation of the routine mastitis prevention and control practices by all of the herd owners. The findings of this study warrants the need for strategic approach including dairy extension that focus on enhancing dairy farmers’ awareness and practice of hygienic milking, regular screening for sub-clinical mastitis, dry cow therapy and culling of chronically infected cows.

**Electronic supplementary material:**

The online version of this article (doi:10.1186/s12917-016-0905-3) contains supplementary material, which is available to authorized users.

## Background

Mastitis is the most widespread and costly disease in dairy cattle occurring throughout the world. It is of particular concern for farmers in developing countries like Ethiopia. Costs due to mastitis include reduced milk production, condemnation of milk due to antibiotic residues, veterinary costs, culling of chronically infected cows and occasional deaths [1]. Moreover, mastitis has a serious zoonotic potential associated with shedding of bacteria and their toxins in the milk [2].

Mastitis is caused by a wide spectrum of pathogens and, epidemiologically categorized in to contagious and environmental mastitis [3]. Contagious pathogens are those for which udders of infected cows serve as the major reservoir. They spread from cow to cow, primarily during milking, and tend to result in chronic sub-clinical infections with flare-ups of clinical episodes. Contagious pathogens include: *Staphylococcus aureus, Streptococcus agalactiae*, *Mycoplasma* spp. and *Corynebacterium bovis* [4]. On the other hand, environmental mastitis can be defined broadly as those intra-mammary infections caused by pathogens whose primary reservoir is the environment in which the cow lives [5]. Environmental pathogens include *E. coli*, Klebsiella spp., *Strept. dysgalactiae* and *Strept. uberis* and the majority of infections caused by these pathogens are clinical and of short duration [6].

Mastitis can also be classified as either clinical or sub-clinical. Clinical mastitis is characterized by sudden onset, alterations of milk composition and appearance, decreased milk production, and the presence of the cardinal signs of inflammation in infected mammary quarters. It is readily apparent and easily detected. In contrast, no visible signs are seen either on the udder or in the milk in case of sub-clinical mastitis, but the milk production decreases and the somatic cell count increases. It is more common and has serious impact in older lactating animals than in first lactation heifers [7]. Because of the lack of any overt manifestation, the diagnosis of sub-clinical mastitis is a challenge in dairy animal management and in veterinary practice [8].

Ethiopia has the largest cattle population in Africa, which is estimated at 53.99 million animals of which 7.2 million are primarily held for milk production [9]. Despite these large numbers, milk production often does not satisfy the country’s requirements due to a multitude of factors amongst which the occurrence of mastitis has great prominence. Over the years a number of researchers have studied the occurrence of mastitis in dairy herds in Ethiopia. According to the most recent published studies, the cow-level mastitis prevalence estimate falls within the range of 23.2 and 81.1% for the country [10–16]. Consequently, the disease has been documented as one of the major constraint of the dairy sector that needs attention.

Most of the previous studies in Ethiopia were concentrated on the investigation of the prevalence and few risk factors for mastitis at cow level and no or little effort has been made to assess the prevalence, management and hygienic practices at herd/farm-level. A focused study on contagious mastitis with emphasis on subclinical type is lacking. Besides, teat morphology, which is inherited anatomical feature of the cow that may affect mastitis occurrence [17] and could serve as a marker trait for selection to reduce mastitis in dairy cattle [18], is less investigated. It is therefore important to assess the effect of the variable on clinical and sub-clinical mastitis under Ethiopian condition and recommend preventive measures to reduce loss attributed to the disease. Moreover, given the huge economic relevance due to lack of clinical visibility and subsequent effects, investigation of sub-clinical mastitis at herd-level is of paramount importance for designing feasible prevention and control strategy.

Thus, this study was aimed to estimate the prevalence of clinical and sub-clinical mastitis, identify the major risk factors and isolate *S. aureus,* one of the etiological agents for contagious mastitis, from dairy cows in intensive and semi intensive management in southern Ethiopia.

## Methods

### Study area

This study was conducted between December 2014 and May 2015 in dairy herds found at Hawassa milk shed. The milk shed includes Hawassa city and its adjacent towns which supply milk for the big market at Hawassa. For this study, Hawassa city and two of the towns (Wondo Genet and Arsi Negelle) that are supplying milk for Hawassa were purposively selected because of their relatively larger potential for dairy cattle population. Hawassa is the capital city of the Southern Nations, Nationalities and Peoples’ Regional State, and located at 275 km south of Addis Ababa, the capital. The city is situated at an elevation of 1708 m above sea level and located at 7^0^ 3’ north latitude and 38^0^ 29’ east longitudes. The annual rain fall and temperature varies from 800 to 1000 mm and 20.1–25 °C, respectively. Wondo Genet is located at 30 km west of Hawassa. It is situated about 1723 m above sea level at latitude of 7^0^5’ N and longitude of 38^0^ 37’ E. The town receives an average annual rainfall of 1372 mm. The mean annual temperature is 19 °C. Arsi Negelle town is found in the West Arsi zone of the Oromia regional state at a distance of 225 km from Addis Ababa. The town is situated about 2043 m above sea level at latitude of 7^0^ 21’ N and longitude of 38^0^ 42’ E. The average annual temperature of the area varies from 10 to 25 °C while rainfall varies between 500 and 1000 mm.

### Study population and farm type

A total of 95 dairy herds selected randomly from the dairy farms present in the study areas were included in the study. The herd size of the selected farms varied from four to 112 cattle of which two to 57 were lactating cows. A total of 529 lactating cows were selected. Most of them (84.3%) were cross-breeds (Holstein-Friesian x Zebu) whilst few were local (Borana and Arsi breeds) (15.7%). With regard to management, 35 (36.8%) of the herds were managed intensively while 60 (63.2%) herds were semi-intensive. The intensively managed cattle were kept in doors and received concentrate feeds in addition to hay and crop residues (such as corn stalks, wheat/barley straw and other leftovers from grain threshing). On the other hand, the semi-intensively managed cattle grazed freely on pasture but received supplementary feeds in the morning and evening when they were milked. All cows were hand milked twice daily, in the morning and evening. The milk yield of the cows ranged from four to 14 l per day for cross breeds while two to four liters for local breeds.

### Study design and sample size

A cross-sectional study design was followed to address the objective of the study. The sample size was estimated following the method described by Thrusfield [19] for simple random sampling with 95% confidence level and 5% absolute precision, considering an optimum expected herd level prevalence of 50%. Accordingly, the sample size was computed to be 384 herds. This sample size would be acceptable if the size of the study population were large in relation to the sample. However, since the number of dairy farms in the region registered and known by the respective district veterinary departments was smaller (only 126) than the calculated sample size, an adjustment was made based on the formula recommended for a small population size [19].

$$ {n}_{\mathrm{adj}}=\frac{N\times n}{N+n} $$

Where n is the sample size based on an infinite population and *N* is the size of the study population. Thus, the required sample size, *n*_adj,_ was calculated to be 95. Consequently, proportional allocation was made for each district in the milk shed and 40 herds each was considered for Hawassa and Arsi Negele and the remaining 15 allotted for Wondo Genet. In each district, respective herd sampling frame was constructed in collaboration with district veterinary department and herds were picked randomly using computer generated random numbers. Before data collection, a support letter written by the respective district veterinary department was sent to the identified farms requesting them to collaborate in the study. Fortunately, all of the farm owners who were randomly selected showed willingness to participate in the study. In each herd a minimum of 10% of the lactating cows were picked at random following the same procedure used for herd selection. Eventually a total of 529 lactating cows were included in the study.

### Data collection

A structured questionnaire with close ended questions was used to collect data about various herd- and animal-level factors thought to influence the occurrence of mastitis in the dairy herds. The herd-level factors assessed were study area, herd size, herd management system, use of bedding material, milking practices (machine/hand), frequency of milking, cleaning and drying methods of udder, post-milking teat dipping, hygiene of cow barns, milking mastitic cow last, dry cow therapy, and culling of chronically infected animals. Those farms with good drainage system and used to clean cow manure daily were considered as “good hygiene” while those with no drainage system and absence of daily manure cleaning was regarded as “poor hygienic status.” For the purpose of this study and ease of data analysis, the herd size was categorized as ≤10 cows and >10 cows. This is due to the fact that under Ethiopian context, farms with ≤10 cows are small scale semi-intensive farms that are run as side business. Moreover, these farms usually do not have feed reserves sufficient for a year and operated mainly by family labor. In contrast, farms with more than ten cows are medium or large scale intensive farms that have feed reserves sufficient for a year, operated by hired labor and owned by farmers whose primary occupation is dairying. Thus, the herds were categorized in that way just to evaluate the effect of herd management difference on mastitis occurrence. Pertinent to cow-level factors, biodata including lactation (age, parity & stage), breed, milk yield, udder and leg hygiene, teat end morphology, udder position, and previous mastitis history were recorded.

The udder and leg hygiene of each cow was assessed and scored based on a four-point scale (1–4) as described by Schreiner and Ruegg [20]. Accordingly, an udder hygiene score (UHS) or leg hygiene score (LHS) of ‘1’ was referred to no contamination of the skin of the rear of the udder or the hind limb between the hock and coronary band; ‘2’ slightly dirty (2–10% of the area covered in dirt; ‘3’ moderately dirty (10–30% of the area covered in dirt); and ‘4’ caked-on dirt (>30% of these areas completely covered in dirt).

Each cow was clinically observed for the manifestation of general clinical signs related to udder and teats and presence of any gross abnormalities like fibrosis, inflammatory swellings, pain, visible injury or lesion, atrophy of the tissue and teat blindness. The milk sample was observed for any change regarding color, odor and consistency. The presence of clots, flakes, blood and other consistency changes were part of the indicators for clinical mastitis along with udder and teat morphological changes. Udder position was recorded as normal or pendulous while teat end shape was categorized as pointed, round or flat. Though all the researchers were involved in questionnaire and data collection format designing, to avoid any bias, all the field data collection process (questionnaire, hygiene scores, CMT, milk sample collection, etc.) was done by one of the researchers. During the study period each farm was visited only once.

### California mastitis test

The California mastitis test (CMT) was used as a screening test for sub-clinical mastitis. It was carried out according to the procedure described by Quinn et al. [21]. A squirt of milk, about 2 ml from each quarter was placed in each of four shallow cups in the CMT paddle. An equal amount of the commercial CMT reagent was added to each cup. A gentle circular motion was applied to the mixtures in a horizontal plane for 15 s. The CMT results were scored as 0 (negative), trace, 1(weak positive), 2 (distinct positive) and 3 (strong positive) based on gel formation. All CMT scores of 0 and trace were considered as negative while CMT scores of 1, 2, and 3 were considered indicators of sub clinical mastitis. Positive cows were defined as having at least one quarter with CMT score of 1 + .

### Isolation and identification of *Staphylococcus aureus*

For identification of the causative microorganisms, milk samples were collected from about 50% of CMT positive cows, randomly selected from all mastitis cases, assuming that the causative organisms within a herd are similar and also to reduce the time, labor and cost burdens. In case where only one mastitis case found in a farm, that positive cow was directly sampled. CMT negative cows (0 and trace scores) were not included in milk sampling. During sampling, milk was collected from all quarters of CMT positive cows at the same time as the CMT was done.

Milk sampling was carried out following aseptic procedures as described by National Mastitis Council (NMC) [22]. The time chosen for milk sample collection was before milking. Udder and especially teats were cleaned and dried before sample collection. Each teat end was scrubbed vigorously with a pledged of cotton moistened with 70% ethyl alcohol. Recontamination of teats during scrubbing was avoided by scrubbing carefully, the teats on the far side of the udder first, then those on the near side. A separate pledged of cotton was used for each teat. Teats towards sample collection were sampled first and then the far ones. The first few streams of milk were discarded and approximately 10 ml of milk was collected into horizontally held sterile universal sample collection bottle. After collection, the samples were labeled and placed in icebox and transported to the microbiology laboratory of the School of Veterinary Medicine, Hawassa University. The samples were immediately cultured or stored at 4 °C for a maximum of 24 h until cultured on standard bacteriological media.

To comply with specific objective of the study, bacteriological examination was focused only on identification and isolation *S. aureus*, which is the most common cause of contagious mastitis in dairy herds worldwide. The isolation of *S. aureus* was carried out according to the standard microbiological procedures described by NMC [22]. One standard loop (0.01 ml) of milk sample was streaked on 7% sheep blood agar (Oxoid, Hampshire, England) using the quadrant streaking method for each cow. In case of refrigerated milk samples, as bacteria might be concentrated in the cream layer and held with in clumps of fat globules, dispersion of fat and bacteria was accomplished by warming the samples at 25 °C for 15 min before plating on blood agar. The inoculated plates were then incubated aerobically at 37 °C for 24 to 48 h. When growth was not observed after incubation for 24 to 48 h, the milk sample was re-inoculated on an enriched tryptone soya broth (Oxoid, Hampshire, England) to amplify the bacterial growth. The plates were examined for growth, morphologic features such as colony size, shape, color and hemolytic characteristics. Presumptive colonies were selected and sub cultured on nutrient agar (Oxoid, Hampshire, England) and incubated aerobically at 37 °C for 24–48 h to get a pure culture. After incubation, colonies were identified according to their Gram reaction (Gram-positive or Gram-negative), cellular morphology (coccus or rod) and arrangements of the bacteria and the catalase test. The Gram and catalase-positive cocci were characterized for mannitol fermentation on mannitol salt agar (Oxoid, Hampshire, England), which was followed by tube coagulase test. Samples were considered positive for *S. aureus* when at least one colony was identified as *S. aureus.*

### Data analysis

The data were recorded in Microsoft Excel spread sheet and coded before statistical analysis. All the statistical analyses were performed using Stata 11 statistical software (StataCorp, 4905 Lakeway Drive, College Station, Texas). The prevalence of mastitis was calculated as the proportion of mastitis-positive cows (clinical and subclinical) against the total number of animals investigated. A herd was denoted as positive for mastitis if at least a single animal with clinical mastitis or CMT positive result was detected. The association between the dependent variable, cow mastitis status (0 = negative and 1 = positive) and categorical independent variables was assessed using univariable logistic regression analyses. The independent variables evaluated were management type, herd size, use of bedding material, milking mastitic cow last, breed, age, parity, stage of lactation, milk yield, udder and leg hygiene, udder position, teat end morphology, udder washing, udder drying after washing, and previous mastitis history. All variables with *p-value* < 0.25 in the initial univariable analysis were checked for multicollinearity using Kruskal gamma statistics and those variables whose gamma value ranged between −0.6 and +0.6 were considered in a multivariable logistic regression analysis. The final model was built in backward stepwise elimination procedure in reference to log-likelihood ratio and wald statistics. Finally the model was assessed for goodness-of-fit using the Hosmer-Lemeshow method and the predictive ability using the receiver operating characteristic (ROC) curve [23]. In this analysis statistical significance was set at *p* < 0.05.

## Results

### Management and hygienic practices in the dairy farms

During the course of the study, data on various management and hygienic practices implemented by the dairy farms were collected from a total of 95 farms. A summary of data on the practices assessed was presented in Table 1.

**Table 1:** Management and hygienic practices implemented by the dairy farms selected for mastitis study at Hawassa milk shed, South Ethiopia

Variable	Category	No. of herds	Distribution (%)
Herd size	≤10 cows	60	63.2
	>10 cows	35	36.8
Management type	Semi-intensive	60	63.2
	Intensive	35	36.8
Housing	Stall barn	23	24.2
	Group barn	72	75.8
Bedding material	Yes	39	41.1
	No	56	58.9
Pre/post teat dipping	Yes	-	-
	No	95	100
Culling chronically infected cows	Yes	-	-
	No	95	100
Dry cow therapy	Yes	-	-
	No	95	100
Udder washing before milking	Whole udder	56	58.9
	Teats only	39	41.1
Drying of udder after washing	Yes	41	43.2
	No	54	56.8
Milking mastitic cow last	Yes	31	32.6
	No	64	67.4
Hand prewashing	With soap	44	46.3
	Without soap	51	53.7
Hygiene of the farm	Good	19	20
	Poor	76	80

### Prevalence of mastitis

Table 2 illustrates the prevalence of clinical and sub-clinical mastitis at herd, cow and quarter level. Based on CMT results and clinical examination, 74.7% of the herds and 62.6% of the cows were positive for mastitis. All quarters of cows (2119) were checked for the presence of gross abnormalities and it was found that 90 (4.3%) teats were blind while 15 (0.7%) of them had various types of lesions. Subsequently, CMT was conducted on milk samples from 2026 quarters out of which 729 (36%) were positive for mastitis. Sub-clinical mastitis was the predominant type of mastitis observed at herd, cow and quarter levels. Of the total cows with subclinical mastitis, 35.2, 49.5 and 15.3% were weak, distinct and strong CMT positives, respectively.

**Table 2:** Prevalence of mastitis in dairy cows at Hawassa milk shed, South Ethiopia

Observation	Overall mastitis				Clinical mastitis	Subclinical mastitis
	No examined	No pos	Prevalence (%)	95% CI	No (%)	No (%)
Herd-level	95	71	74.7	64.5 – 82.8	10 (10.5)	71 (74.7)
Cow-level	529	331	62.6	58.3 – 66.7	18 (3.4)	313 (59.2)
Quarter-level	2026	729	36	33.9 – 38.1	26 (1.3)	703 (34.7)

### Mastitis risk factors

The prevalence of mastitis at each udder quarter has been shown in Table 3. Overall, the hind quarters were more affected than the fore quarters. The proportion of right hind quarters with positive CMT result was significantly (*p* < 0.001) higher than that of left or right fore quarters but no significant difference was noted between left and right hind quarters (*p* > 0.05).

**Table 3:** Analysis of the prevalence of mastitis in dairy cows at quarter-level

Quarter	No. examined	No. positive	Prevalence (%)	*χ* ^2^	*p*
Right hind	504	249	49.4	Ref	
Left hind	504	223	44.2	2.7	0.101
Right fore	507	132	26.0	59.0	<0.001
Left fore	511	125	24.5	67.6	<0.001

Several herd and cow-level factors described in the data analysis section were considered in the univariable logistic regression analysis for the presence of mastitis. Among those factors, management type, herd size, use of bedding material, milking mastitic cows last, breed, age, parity, stage of lactation, milk yield, udder and leg hygiene, udder position, and teat end morphology were found to be significantly (*p* < 0.05) associated with the presence of mastitis. On the other hand, udder washing, udder drying after washing and previous mastitis history did not have significant effect (*p* > 0.05) on the occurrence of mastitis (Table 4).

**Table 4:** Univariable logistic regression analysis of the association of cow-level mastitis with different risk factors

Variable	Number	No. pos	Prevalence (%)	*p*
Management type				<0.001
Semi-intensive	200	92	46.0	
Intensive	329	239	72.6	
Herd size				<0.001
≤ 10	200	92	46.0	
> 10	329	239	72.6	
Bedding material				<0.001
Yes	145	61	42.1	
No	384	270	70.3	
Udder washing				0.707
Whole udder	291	180	61.9	
Teats only	238	151	63.5	
Udder drying after washing				0.774
Yes	295	183	62.0	
No	234	148	63.3	
Milking mastitic cow last				0.001
Yes	202	109	54.0	
No	327	222	67.9	
Breed				<0.001
Local	83	33	39.8	
Cross	446	298	66.8	
Age				<0.001
≤ 5 years	227	85	37.4	
> 5 years	302	246	81.5	
Parity				<0.001
1	66	17	25.8	
2	145	60	41.4	
3	115	75	65.2	
≥ 4	203	179	88.2	
Stage of lactation				<0.001
1 – 2 months	198	158	79.8	
3 – 6 months	186	80	43.0	
≥ 7 months	145	93	64.1	
Udder & leg hygiene score				<0.001
Slightly dirty	241	120	49.8	
Moderately dirty	203	136	67.0	
Very dirty	85	75	88.2	
Udder position				<0.001
Normal	315	162	51.4	
Pendulous	214	169	79.0	
Teat end shape				<0.001
Pointed	175	74	42.3	
Round	197	126	64.0	
Flat	157	131	83.4	
History of mastitis				0.174
No	189	111	58.7	
Yes	340	220	64.7	

Among the factors considered in the initial univariable analysis, age of the cows was dropped from further analysis due to multi-collinearity with parity (gamma = 0.99), udder position (gamma = 0.97) and previous history of mastitis (gamma = 0.91). With regard to mastitis, parity was considered the more relevant variable than age based on biological plausibility and thus retained. Also milk yield was excluded due to multi-collinearity with herd size (gamma = 0.63) and breed (gamma = 1.00). Likewise, udder position and previous history of mastitis were eliminated from further analysis due to collinearity with parity (gamma = 0.99 and 0.92, respectively). All the cows with four or more parities had pendulous udder and previous history of mastitis. Thus, the variables subjected to multivariable logistic regression analysis were management type, herd size, breed, use of bedding material, milking mastitic cows last, parity, stage of lactation, udder & leg hygiene, and teat end shape. Accordingly, the final logistic regression model revealed that with the exception of management type (*p* > 0.05) all the variables entered remained significant predictors of mastitis in the cows (*p* < 0.05). The Hosmer-Lemeshow goodness-of- fit test suggested that the model fit the data (*χ* ^2^ = 1.7; *p* = 0.99) (Table 5).

**Table 5:** Multivariable logistic regression analysis of the association of cow-level mastitis with different risk factors

Variable	Category	OR	SE	z	*p*	95% CI for OR
Herd size						
	≤10 cows	Ref				
	>10 cows	3.1	1.0	3.74	<0.001	1.7 – 5.7
Breed						
	Local	Ref				
	Cross	16.4	8.3	5.55	<0.001	6.1 – 44.1
Milking mastitic cow last						
	Yes	Ref				
	No	2.0	0.6	2.53	0.011	1.2 – 3.5
Bedding material						
	Yes	Ref				
	No	2.8	0.9	3.24	0.001	1.5 – 5.3
Parity						
	1	Ref				
	2	1.2	0.5	0.48	0.629	0.5 – 2.9
	3	3.2	1.5	2.46	0.014	1.3 – 8.1
	≥4	24.8	12.2	6.50	<0.001	9.4 – 65.2
Stage of lactation						
	Early (1 – 2 months)	Ref				
	Mid (3 – 6 months)	0.3	0.1	−4.17	<0.001	0.1 – 0.5
	Late (≥7 months)	0.7	0.2	−0.99	0.323	0.4 – 1.4
Udder & leg hygiene						
	Slightly dirty	Ref				
	Moderately dirty	2.6	0.8	3.27	0.001	1.5 – 4.7
	Very dirty	8.7	4.0	4.73	<0.001	3.5 – 21.3
Teat end shape						
	Pointed	Ref				
	Round	3.2	1.0	3.72	<0.001	1.7 – 5.9
	Flat	7.6	2.8	5.57	<0.001	3.7 – 15.7
Constant				−7.44	<0.001	

### Bacterial isolation

During the course of the study, a total of 172 milk samples collected from 18 clinical and 154 sub-clinical mastitic cows in 71 herds were cultured on blood agar media. Accordingly, growth of different groups of bacteria was observed in 170 (98.8%) samples. However, since the objective was to isolate *S. aureus*, further bacteriological tests were conducted by taking only those colonies presumptive of this species and leaving others. Finally, *S. aureus* was detected in 51.2% (88/172) of the milk samples cultured. The proportion of isolation from milk of clinically and sub-clinically affected cows was 22.2 and 54.5%, respectively. At herd level, *S. aureus* was isolated from 73.2% of the herds affected with mastitis (Table 6).

**Table 6:** Isolation of *S. aureus* in mastitic milk samples from dairy herds at Hawassa milk shed

Clinical mastitis		Sub-clinical mastitis		Total cultured	Total positive (%)
No. samples cultured	No. pos (%)	No. samples cultured	No. pos (%)		
18	4(22.2%)	154	84(54.5%)	172	88 (51.2%)

## Discussion

This is one of the few bovine mastitis studies in Ethiopia which involved a large sample size of lactating cows (*n* = 529) selected from 95 dairy herds. The study revealed that 74.7% of the herds observed had atleast a cow suffering from mastitis. Due to lack of similarly designed studies with in Ethiopia, it was difficult to compare the herd-level results with other mastitis studies. However, in line with similar studies abroad, the prevalence reported is considerably higher than that of Tanzania (21.7%) [24], Zimbabwe (49.3%) [25] and South Wales (29%) [26].

The overall cow-level prevalence was 62.6% and it was largely accounted from sub-clinical mastitis (59.2%) and the smallest proportion (3.4%) from clinical mastitis. The present finding is within the range of cow-level mastitis prevalence (23.2–81.1%) recorded by most recent published studies in the country [10–16]. Some of the earlier findings and our observation is higher relative to the available reports from other African countries whose dairy management is more or less similar to ours, i.e., 51.6% in Tanzania [24], 42.9% in Egypt [27], 38.89% in Sudan [28], 21.1% in Zimbabwe [25], and 51.8% in Rewanda [29]. In contrast, substantially higher prevalence was reported from Uganda (87.9%) [30] and Nigeria (85.3%) [31]. This entails how serious the problem is, in the dairy industry sector of the continent that warrant due attention.

Indeed both the herd- and cow-level mastitis prevalence observed in the current study are very high. According to Levesque [32], within-herd mastitis prevalence of 40% or over must sound an alarm to the producer. Thus, it is obvious that the high mastitis prevalence seen in the current study area is a serious problem that not only reduces milk production but adversely affects the quality of milk and leads to economic losses and public health hazard. Perhaps the prevailing high prevalence of mastitis could either be due to lack of implementation of regular mastitis prevention and/or control strategies other than treating clinical cases. The fact that none of the dairy herds implementing routine mastitis prevention practices such as post-milking teat disinfection, wearing of gloves during milking, treatment of mastitis during non-lactating period, culling of chronically infected animals and others substantiate the assumption. Moreover, none of the farmers were doing CMT or other tests routinely to screen their cows for sub-clinical mastitis. On the other hand, the risk factors contributing to mastitis were more predominant. These were large herd size, cross breeding local with exotic breeds, not milking mastitic cows last, absence of bedding material, a higher proportion of multiparous older cows with 4 or more parities, early stage of lactation, dirty udder and leg, and teat end morphology,

The finding of sub-clinical mastitis as the predominant form both at cow (59.2%) and herd level (74.7%) is a very clear evidence of huge economic loss the sector is suffering from. In fact, our finding corroborate with earlier reports of 54.4 to 73.3% [11, 14, 15]. Likewise, the preponderance of sub-clinical mastitis and its serious economic relevance compared to clinical mastitis was underscored elsewhere [8, 25, 27, 33, 34]. According to Seegers et al. [1], the sub-clinical form is 15 to 40 times more prevalent than the clinical form, and usually precedes the clinical form and is of long duration and high economic relevance. Thus, it is important to emphasize that the sub-clinically affected animals remain a continuing source of infection for herd mates. The dominance of sub-clinical mastitis in the herds is most likely attributed to the little attention paid by the farmers to this form of mastitis, as the infected animal shows no obvious clinical symptoms and secrets apparently “normal” milk. Lack of implementation of regular mastitis monitoring program such as CMT or other testing by all of the farms also contributed to the observed high prevalence of sub-clinical mastitis in our case. Perhaps this fact may justify the lack of awareness on the invisible losses from sub-clinical mastitis.

The observation of higher mastitis prevalence in the hind quarters is similar to earlier studies [10, 16, 34]. Although it is difficult to give a plausible explanation for this observation, this might be due to the lower position of the hind quarters in relation to fore ones, which make them more prone to contamination or injuries leading to mastitis. Greater milk yield produced by the hind quarters may also be a reason as risk of mastitis tends to increase with increase in milk production [35]. In contrast to the present finding, a significantly higher prevalence was reported in fore than hind quarters in Nigeria [31].

In the present study, the presence of mastitis was significantly influenced by herd size. The odds of finding a cow with positive CMT result was 3.1 times higher in herds with more than ten cows than in herds with ten or fewer cows. It was observed that all the herds with more than ten cows were intensively managed and thus, the higher odds of mastitis occurrence might be associated with increased exposure of the cows for mastitis pathogens in their environment due to high stocking density, dirty ground, infected utensils, poor ventilation and high humidity. The study further revealed that lack of bedding material in the cow barns was significantly associated with the presence of mastitis. Cows in herds that did not use bedding material were 2.8 times more likely to have mastitis than those that did use. It appeared that the floor was a potential source for mastitis organisms, particularly the environmental pathogens, to enter the udder through the teat orifice. In connection to this, Radostitis et al. [4] stated that the use of adequate amounts of good bedding material will reduce incidence of mastitis. In the current study however, no attempt was made to isolate environmental pathogens and thus, difficult to explain what proportion of the herds had these pathogens. Hence, this is considered as one of the limitations of the study that should be addressed in future studies.

Cows in herds that did not milk mastitic cows last were significantly more likely to have mastitis than those that did that. It is obvious that, failure to milk mastitic cows last would favor spread of mastitis pathogens between cows by milker’s hands resulting in contagious mastitis [8]. It is only 31 of the 95 herds that practiced milking mastitic cows last. The current finding complies with reports of Belayneh et al. [12] in Ethiopia.

Increasing parity number was also one of the predictors noted to associate with the presence of mastitis. Such scenario has also been documented in a number of studies [12, 14, 25, 30, 36]. The likelihood of mastitis was 24.8 times higher in multiparous cows having four or more calvings compared with primiparous cows. This partly, might be associated with the position of udder in older cows. In the current study it was noticed that all of the older cows particularly those with four or more parities had pendulous udder and previous history of mastitis. It has also been stated that cows with the most pendulous quarters appear to be the most susceptible to mammary infections, the pendulous udder exposes the teat and udder to injury and pathogens easily adhere to the teat and gain access to the gland tissue [34].

Cows in the early lactation were significantly less likely to have mastitis than cows in the mid lactation stage albeit no significant difference was appreciated between early and late lactation stages. Evidence on the trend of prevalence increment at the early stage was also produced in previous studies [14, 16, 27, 29]. Perhaps this could be linked to the fact that diapedesis of neutrophils into the mammary gland take longer time in recently calved cows [4], and increased oxidative stress and reduced antioxidant defense mechanisms during early lactation [37]. Moreover, absence of dry cow therapy regime could possibly be among the major factors contributing to higher prevalence at early lactation. In contrast to the present finding, Belayneh et al. [12] reported higher prevalence of mastitis in late stage of lactation while Mureithi and Njuguna [36] in Kenya found a significantly higher prevalence in the mid stage. The variations in the effect of stages of lactation among different studies could be related to disparities in age, parity and breed of the sampled animals. However, the actual source of variation needs to be sorted out in the future research.

The status of cows’ udder and leg hygiene was also noted as part of the risk factors that enhance the occurrence of mastitis. Based on the udder and leg hygiene score used, all the examined cows had slightly to very dirty udders and legs, and there was a marked increase in the detection of mastitis as the level of dirtiness increases. Thus, the odds of mastitis presence was 2.6 or 8.7 times higher in cows with moderate or very dirty udder and legs compared to cows with slightly dirty udder and legs. This dirtiness of cows’ udder and legs was the result of poor hygiene of the dairy farms. Of the total farms investigated, 80% were categorized as poor hygienic due to lack of waste drainage system and accumulation of manure and urine. In agreement to the present study, a significant association between poor udder hygiene and increased risk of mastitis has also been reported by other studies [29, 30, 36].

In this study, a strong association was observed between teat end shape and CMT positive result. Cows with flat teat or round teat ends were 7.6 or 3.2 times more likely to have mastitis than cows with pointed teat ends. This result may be explained by the presence of wider streak canals in teats with rounded or flat ends, which have been shown to allow more pathogen penetration [38]. The current finding is also in agreement with Bharti et al. [39] who reported a significantly higher mean somatic cell counts in cows with flat teat-end shape.

This study also revealed a significant association between breed and the presence of mastitis. The likelihood of mastitis was 16.4 times higher in Holstein-Friesian x zebu crosses than pure local zebu cattle. This shows that pure local breeds are more resistant to contracting mastitis than the European. Breed difference in susceptibility to mastitis has also been reported by other studies [29, 31, 40].

According to the microbiological finding of the study, *S. aureus* was isolated from 51.2% mastitic milk samples and 73.2% herds affected with mastitis. The cow-level finding is comparable to a previous report of 51.7% from northern part of Ethiopia [16] and also other mastitis studies in the country which recorded *S. aureus* as the predominant agent [11–15]. Apart from Ethiopia, *S aureus* has also been reported as the chief aetiological agent of mastitis in cattle by many studies from African and Asian countries [8]. The high prevalence of *S. aureus* in this study could be associated with absence of post milking teat dipping, lack of culling of chronically infected cows, absence of dry cow therapy and the invariable hand milking practice among the dairy herds. *S. aureus* and other contagious microorganisms are usually found on the udder or teat surface of infected cows and are the primary source of infection between uninfected and infected udder quarters, usually during milking. Even though in all the observed herds milkers’ used to wash their hands before milking, they do it only before milking the first cow. Hence it is evident that the causative organisms could be transmitted easily from infected to uninfected udder quarters or cows through the milkers’ hands. Cure rate of *S. aureus* infections with antibiotic therapy during lactation is very low and many infected animals become chronic cases and have to be culled [41]. Thus the fight against contagious mastitis should encompass dry cow therapy as major component of mastitis control program. Unfortunately, none of the dairy farmers have the practice of culling of chronically infected animals, dry cow therapy and post-milking teat disinfection and this has created a good opportunity for the establishment of the organism in the dairy herds.

## Conclusions

The present study has shown that mastitis, particularly sub-clinical type, is a widely prevalent disease in the dairy farms of Hawassa milk shed at both herd- and cow-level. Lack of implementation of the routine mastitis prevention and control practices by all of the farms observed and preponderance of the risk factors noted are the main reasons for the observed high prevalence of mastitis in the milk shed. Accordingly, multiparous cross-breed cows at early stage of lactation which had dirty udder and legs and round or flat teat ends were at higher risk of contracting mastitis. Besides, larger herds, failure to milk mastitic cows last and keeping cows in the absence of bedding material were also noted to increase the exposure of cows for mastitis pathogens. The present study has also revealed that *S. aureus* is an important cause of mastitis which was isolated from more than half of mastitic cows tested and 73.2% of herds affected with mastitis. Moreover, the isolation of *S. aureus* in observed proportion indicates the higher public health risk due to consumption of raw milk and its products.

Therefore the current study warrants the need for applying feasible mastitis intervention strategy with special emphasis on contagious and sub-clinical mastitis. These include strong dairy extension service that focused on awareness creation and hygienic milking practice. Besides, the animal health service delivery need to focus on regular screening of dairy cows for subclinical mastitis and treating of the cases both in lactation and dry period, and provision of advice to cull chronically infected animals. Since the current study on etiological agents was limited to contagious ones, future studies in the milk shed should include isolation of environmental pathogens. Last but not least the rational use of antibiotic and regular antibiogram surveillance should be made part and parcel of the approach.

## Additional file

Additional file 1: Raw data from bovine mastitis study at Hawassa milk shed, South Ethiopia. The additional file contains the entire herd and animal-level raw data collected from 529 lactating cows at Hawassa milk shed, South Ethiopia and on which the conclusion of the manuscript rely. The data will be freely available at the journal’s webpage to any scientist wishing to use them for noncommercial purposes. (XLSX 86 kb)
